# Association between self-efficacy, spiritual well-being and the willingness to provide spiritual care among nursing staff in Taiwan: a cross-sectional study

**DOI:** 10.1186/s12912-024-01978-x

**Published:** 2024-04-30

**Authors:** Shu-Hui Yang, Yu-Tse Tsan, Wan-Ting Hsu, Chin-Feng Liu, Wen-Chao Ho, Li-Fen Wu, Cheng-Fu Lin, Wei-Min Chu

**Affiliations:** 1 Department of Nursing, Taichung Veterans General Hospital, Taichung, Taiwan; 2 Department of Occupational Medicine, Taichung Veterans General Hospital, Taichung, Taiwan; 3 Department of Family Medicine, Taichung Veterans General Hospital, Taichung, Taiwan; 4 School of Public Health, China Medical University, Taichung, Taiwan; 5 Department of Nursing, National Taichung University of Science and Technology, Taichung, Taiwan; 6 Center for Geriatrics & Gerontology, Taichung Veterans General Hospital, Taichung, Taiwan; 7 Geriatrics and Gerontology Research Center, College of Medicine, National Chung Hsing University, Taichung, Taiwan; 8 Department of Post-Baccalaureate Medicine, College of Medicine, National Chung Hsing University, Taichung, Taiwan; 9 School of Medicine, National Yang Ming Chiao Tung University, Taipei, Taiwan; 10 School of Medicine, Chung Shan Medical University, Taichung, Taiwan; 11 Department of Epidemiology on Aging, National Center for Geriatrics and Gerontology, Aichi, Japan

**Keywords:** Spiritual care, Willingness, Nurse, Self-efficacy, Spiritual well-being

## Abstract

**Background:**

Spiritual care plays a significant role in holistic patient care, addressing not only physical ailments but also attending to patients’ emotional and spiritual well-being. While the importance of spiritual care in nursing is widely recognized, there is often a gap in understanding nurses’ willingness to provide such care. This cross-sectional study aimed to explore the association between self-efficacy, spiritual well-being, and willingness to provide spiritual care among nursing staff.

**Methods:**

The study conducted a cross-sectional survey of full-time registered nurses at a hospital in Taiwan from January 2019 to December 2019. A sample comprising 168 nurses was selected for participation in the study through a random sampling method. In addition to collecting demographic variables, the assessment tools used in the study include the General Self-Efficacy Scale (GSES) for measuring self-efficacy, the Spiritual Index of Well-Being Chinese Version (SIWB-C) for evaluating spiritual well-being, and the Spiritual Care Needs Inventory (SCNI) to gauge willingness to provide spiritual care.

**Results:**

Most participants in the study were female, accounting for 98.2% (*n* = 165). The mean age of all 168 nurses was 37.1 ± 9.3 years. Additionally, most participants held a Bachelor’s degree (79.2%, *n* = 133) and possessed clinical experience was 10.5 ± 9.3 years. Through logistic regression analysis, it was found that regardless of whether participants have received sufficient spiritual care training, both GSES and SIWB-C remain influential factors in determining the provision of spiritual care.

**Conclusions:**

Collaboration between healthcare management and nursing staff is essential for fostering a healthcare environment that not only appreciates the physical and spiritual dimensions of patient care but also prioritizes the enhancement of nurses ' self-efficacy and well-being.

## Background

The global acceleration of aging highlights the recent emphasis on healthcare professionals providing spiritual care. Spirituality, challenging to define, represents a dynamic dimension of human nature seeking ultimate meaning, purpose, and transcendence, influencing relationships with oneself, family, community, and more [1]. Increasing evidence suggests that spiritual care contributes to overall healthcare improvement. A systematic review links higher engagement in spiritual activities to reduced mortality risk, substance use, and positive associations with quality of life and psychological well-being [2]. The Joint Commission on Accreditation of Healthcare Organizations recognizes spirituality assessment as essential in healthcare facility evaluations [3]. Among healthcare professionals, nurses play a crucial role, particularly in providing spiritual care for terminally-ill patients, acting as a link between different levels of care and ensuring high-quality patient care [4, 5]. Holistic nursing care focuses on addressing the spiritual care needs of patients along with their physical, emotional, and mental health [6]. Nurses’ spirituality significantly enhances patients’ spiritual well-being, enabling healthcare professionals to deliver more comprehensive and compassionate care through integration into nursing practice [7].

Self-efficacy, a fundamental construct within the framework of social cognitive theory, pertains to an individual’s conviction regarding their capacity to exert control over the circumstances that unfold during their life. It encompasses a profound sense of confidence in one’s ability to overcome obstacles and navigate challenges successfully [8]. Nurses display differing levels of proficiency in different aspects of spiritual care, with competence linked to factors like self-efficacy, training, experience with terminally ill patients, duration of work, and attainment of a bachelor’s degree in nursing [9]. Additionally, providing spiritual care education to nursing students has been shown to enhance their self-efficacy [10]. Another important factor related to spiritual care is the spiritual well-being felt among nurses. Nurses’ spiritual care can be increased via the development of specific strategies focused on enhancing the nursing workplace spirituality of hospital organizations, promoting individual spiritual well-being and compassionate behavior, and reducing burnout among nurses [11]. Nurses who demonstrate higher levels of proficiency in providing spiritual care also tend to experience enhanced spiritual well-being themselves [12]. Moreover, the propensity of nurses to provide religious aspects of spiritual care interventions was not notably tied to their religious beliefs but rather correlated with their personal spiritual well-being [13].

The crucial role of spirituality in improving healthcare outcomes is gaining recognition, supported by empirical evidence linking spiritual practices to reduced mortality risk, improved quality of life, and enhanced psychological well-being. However, more understanding is needed on how healthcare professionals, especially nurses, can effectively provide spiritual care. The study aims to analyze the factors influencing nurses’ self-efficacy and spiritual well-being in providing spiritual care for patients.

## Methods

### Study setting and sampling

The present research employed a cross-sectional survey design utilizing simple random sampling in order to collect data from eligible full-time registered nurses employed at a hospital in Taiwan from January 2019 to December 2019. The nursing staff employed at a tertiary medical center in central Taiwan, who all hold a valid nursing license, were selected as the study participants. A total of 2006 nurses were included in the pool, from which 168 nurses were randomly sampled through a computer-generated randomization table. The research methodology and objectives were explained by the principal investigator during both the morning meetings and shift handovers. Participants were offered the right to refuse participation without there being any future negative impact on their personal advancement. After completing the questionnaires on the same day, they were sealed in provided envelopes and collected by the principal investigator. To avoid disclosure of names and characteristics of the participants, we de-identified our collected data, and the primary investigator is different from the researcher who performed the statistical analysis.

### Data collection and process

The questionnaire utilized in this study aimed to gather information regarding the participants’ basic characteristics and their willingness to provide specific aspects regarding spiritual care. The basic characteristics included age, gender, level of education, department, religious beliefs, participation in an educational program about spiritual care, and the total number of years in nursing practice. To assess the self-efficacy among participants, the general self-efficacy scale (GSES) was employed [14]. A 17-item questionnaire was employed, utilizing a 6-point Likert scale for participant responses. The scale ranged from 0 to 5, with 0 indicating “strongly disagree,” 1 denoting “disagree somewhat,” 2 representing “disagree a little,” 3 for “agree a little,” 4 corresponding to “agree somewhat,” and 5 aligning with “strongly agree.” The total score obtained was 85, with a higher score indicating a stronger belief in an individual’s ability to handle various tasks and challenges in life. This scale had been previously validated in a study with internal consistency being 0.81 [14]. The Spiritual Index of Well-being (SIWB) was designed to gauge individuals’ perceptions of their spiritual quality of life. The original questionnaire demonstrated strong internal consistency, boasting an alpha coefficient of 0.91, and commendable test-retest reliability of 0.79 after a two-week interval [15]. It has been effectively employed in various research studies involving general outpatient patients, chronic patients, older adults, and terminally ill patients [15]. Post-translation and validity assessments conducted by the researchers revealed that the Chinese version of the SIWB (SIWB-C) exhibited robust internal consistency, with a Cronbach’s alpha coefficient of 0.94 in elderly Taiwanese [16]. In this study, we focused on assessing nurses, with the consistency coefficient for the self-efficacy domain being 0.85, for the life scheme domain being 0.92, and for the total scores being 0.92. The SIWB-C questionnaire comprised two domains: six items assessed the intrapersonal self-efficacy domain, while another six items measured the life scheme domain. Utilizing a self-administered approach, respondents rated their agreement on a 5-point Likert scale, ranging from 1 (strongly disagree) to 5 (strongly agree). The questionnaire was reverse-scored, and the total score obtained was 60, where higher scores indicated lower levels of spiritual well-being.

The original spiritual care needs inventory (SCNI) had been previously developed and validated in a study involving 1,351 adult acute care patients recruited from a medical center in Taiwan [17]. The item-level content validity index (CVI) for the SCNI ranged from 0.82 to 1.00, with an instrument-level CVI of 0.87. Additionally, the SCNI demonstrated strong internal consistency with a Cronbach’s alpha coefficient of 0.96. Through principal component analysis, two distinct factors were identified and labeled as “caring and respecting” and “meaning and hope” [17]. In the present study, a modified 20-item SCNI was utilized. The response categories in the original patient version of the SCNI were adjusted for the nurse’s version to assess ‘willingness’ (willing, don’t know how to provide, or unwilling) rather than ‘needs’ (need, neutral, or do not need). The total score of 100 in the nurse’s version of the SCNI, with higher scores signifying a greater willingness for spiritual care, demonstrated a Cronbach’s alpha coefficient of 0.95 among the participants. Results from a pilot study suggested that participants could complete the entire questionnaire in approximately 15 min [18]. In this study, participants were divided into two groups based on their willingness to provide spiritual care. The group willing to provide spiritual care consisted of participants who answered “willing” or higher on all 20 items of the questionnaire. The group unwilling to provide spiritual care consisted of participants who answered “neutral” or lower on at least one item.

### Data analysis

The analysis utilized non-parametric tests due to the data’s deviation from a normal distribution by SAS 9.4 software. Continuous variables were presented as mean ± standard deviation (SD), while categorical data were expressed as number and percentage. The Spearman’s correlation tests was utilized to investigate the relationships between age, work experience, self-efficacy, spiritual well-being and willingness to provide spiritual care. Logistic regression was used to predict the willingness to provide spiritual care. Finally, subgroup analysis was conducted using logistic regression, stratified by self-perceived inadequate training in spiritual care. In the post hoc power analysis, G-Power was used for sample estimation. The parameters for the t-test in the life scheme domain showed that the willing group had 49 participants with a mean of 10.4, while the unwilling group had 119 participants with a mean of 12.8. The SD was 4.9, and the desired power level was set at 81.8%. The statistical analysis was performed using SAS 9.4 statistical software. A *P*-value of less than 0.05 was considered statistically significant.

## Results

Table 1 showed the general characteristics of all participants. Among the 168 participants, most were female (98.2%), had graduated from college (79.2%), worked in an ordinary ward (61.9%), possessed no religious beliefs (38.1%). The mean age was 34.0 37.1 ± 9.3 years and working years was 10.5 ± 9.3 years. GSES had 60.4 ± 10.9, SIWB-C had 24.5 ± 8.0, and SCVI had 81.0 ± 9.9. 155 participants (92.3%) felt that they had not received sufficient training regarding spiritual care.

**Table 1:** Characteristics of the participants (*N* = 168)

Variables	*N* (%)/Mean ± SD	Minimum	Maximum	
Age (year)	37.1 ± 9.3	26.0	63.0	
Gender				
Male	3 (1.8%)			
Female	165 (98.2%)			
Educational level				
Junior college	14 (8.3%)			
College	133 (79.2%)			
Master’s degree	21 (12.5%)			
Department				
Clinic	23 (13.7%)			
Medical/ Surgical Ward	104 (61.9%)			
Emergency Room	1 (0.6%)			
Intensive Care Unit	4 (2.4%)			
Case Management Team	18 (10.7%)			
Other^†^	18 (10.7%)			
Religious belief				
No	64 (38.1%)			
Buddhism	25 (14.9%)			
Taoism	29(17.3%)			
Christianity	4 (2.4%)			
Folklore religion	41 (24.4%)			
Others^‡^	5 (3.0%)			
Taken a course on “Spiritual Care” at school				
Yes	82 (48.8%)			
No	86 (51.2%)			
Taken a course on “Spiritual Care” during post-graduation nursing continuing education				
Yes	81 (48.2%)			
No	87 (51.8%)			
Feel that I have received sufficient training on spiritual care				
Yes	13 (7.7%)			
No	155 (92.3%)			
Work experience (year)	10.5 ± 9.3	1.0	37.0	
<10	101 (60.1%)			
≧10	67 (39.9%)			
General self-efficacy scale	60.4 ± 10.9	30.0	85.0	
Chinese version of Spiritual Index of Well-being	24.5 ± 8.0	12.0	47.0	
Self-efficacy domain	12.4 ± 4.0	6.0	23.0	
Life scheme domain	12.1 ± 4.9	6.0	24.0	
Spiritual care needs inventory	81.0 ± 9.9	65.0	100.0	

^**†**^ respiratory care center, hospice ward

^**‡**^ I-Kuan Tao, Tenrikyo, Mormonism, Jehovah’s Witnesses

Table 2 categorized the willingness of each item on the SCNI into groups. The willing group, consisting of 49 participants, rated “willing” or higher on all 20 items, while the unwilling group, comprised of 119 participants, rated “neutral” or lower on at least one item. The significant correlations between SCNI and age, work experience, GSES, and SIWB-C (all *p*-values < 0.05).

**Table 2:** Correlation of willingness to provide spiritual care with age, work experience, self-efficacy, and spiritual well-being

Variables	Spiritual Care Needs Inventory	
	*r*	*p* value
Age	0.28	0.0003***
Work experience (year)	0.25	0.0011**
General self-efficacy scale	0.37	< 0.0001***
Chinese version of Spiritual Index of Well-being	-0.38	< 0.0001***
Self-efficacy domain	-0.22	0.0039**
Life scheme domain	-0.44	< 0.0001***

Spearman’s correlation tests. *p*-value: * 0.05, ** 0.01, *** 0.001

Table 3 presented logistic regression results indicating that the GSES and SIWB-C, and the Life scheme domain significantly influenced willingness to provide spiritual care, among other factors.

**Table 3:** Willingness to provide spiritual care

Variables	OR	95% Cl	*p*-value
Age (year)	1.02	(0.989, 1.060)	0.1885
Gender			
Male	REF		
Female	0.82	(0.073, 9.263)	0.8729
Educational level			
Junior college	REF		
College	1.58	(0.417, 5.956)	0.5021
Master’s degree	1.47	(0.299, 7.184)	0.637
Department			
Clinic	REF		
Ward	0.63	(0.315, 1.240)	0.1791
Religious belief			
No	REF		
Yes	1.59	(0.781, 3.223)	0.2018
Taken a course on “Spiritual Care” at school			
Yes	1.13	(0.582, 2.205)	0.713
No	REF		
Taken a course on “Spiritual Care” during post-graduation nursing continuing education			
Yes	1.48	(0.757, 2.883)	0.2527
No	REF		
Feel that I have received sufficient training on spiritual care			
Yes	2.23	(0.710, 7.022)	0.1694
No	REF		
Work experience (year)			
<10	REF		
≧10	1.34	(0.683, 2.629)	0.3948
General self-efficacy scale	1.04	(1.005, 1.071)	0.0244*
Chinese version of Spiritual Index of Well-being	0.94	(0.897, 0.984)	0.0077**
Self-efficacy domain	0.92	(0.844, 1.007)	0.0725
Life scheme domain	0.89	(0.821, 0.963)	0.0037**

Logistic regression. *p*-value: * 0.05, ** 0.01

Table 4 listed the results of subgroup analysis for 155 participants, emphasizing that perceived inadequate training in spiritual care can reduce the willingness to provide it. GSES, SIWB-C, and the life scheme domain emerged as notable influencers.

**Table 4:** Inadequate training in spiritual care can diminish one’s willingness to provide it (*n* = 155)

Variables	OR	95% Cl	*p*-value
Age (year)	1.01	(0.975, 1.052)	0.5168
Gender			
Male	REF		
Female	0.38	(0.023, 6.192)	0.4957
Educational level			
Junior college	REF		
College	2.2	(0.472, 10.620)	0.3105
Master’s degree	2.2	(0.371, 13.038)	0.3851
Department			
Clinic	REF		
Ward	0.69	(0.333, 1.411)	0.3051
Religious belief			
No	REF		
Yes	2.18	(1.000, 4.762)	0.0501
Taken a course on “Spiritual Care” at school			
Yes	0.85	(0.419, 1.724)	0.653
No	REF		
Taken a course on “Spiritual Care” during post-graduation nursing continuing education			
Yes	1.10	(0.545, 2.227)	0.788
No	REF		
Work experience (year)			
<10	REF		
≧10	1.11	(0.544, 2.274)	0.7696
General self-efficacy scale	1.04	(1.005, 1.075)	0.0251*
Chinese version of Spiritual Index of Well-being	0.95	(0.901, 0.991)	0.0194*
Self-efficacy domain	0.93	(0.849, 1.022)	0.1342
Life scheme domain	0.90	(0.826, 0.974)	0.0094**

Logistic regression. *p*-value: * 0.05, ** 0.01

## Discussion

The study emphasizes the indispensable role of nurses in addressing spiritual care, as well as the relevant influencing factors affecting the provision of spiritual care. Our study highlights the significant influence of the GSES and the SIWB-C in facilitating spiritual care provision, regardless of variations in spiritual care training. This underscores the necessity for comprehensively understanding and integrating these factors into practice.

The results of the recent study highlight that spiritual care competence is influenced by various factors [19]. Recent studies suggest that younger nurses may exhibit a greater willingness to conduct spiritual assessments [18]. Conversely, according to other research [20], factors such as age, clinical experience, gender, education level, and personal religiosity do not influence the willingness to deliver spiritual care. Notably, nurses who perceive sufficient training in spiritual care delivery tend to display a higher readiness to provide such care to patients [20]. In our study, willingness to provide spiritual care was found to be correlated with age, work experience, GSES, and SIWB-C. However, the main influencing factors were identified as GSES and SIWB-C.

There’s a positive correlation between the number of years of work experience as a registered nurse and spiritual care competence [19]. Additionally, individuals who received education in spiritual care showed notable improvements in competence [19]. Consistent with prior research, our study identified a correlation between work experience and SNCI, implying that this association may be influenced by the accumulation of practical knowledge and skills over time, as well as the potential impact of formal education on enhancing spiritual care competence.

Differentiate self-efficacy from actual skills or competence, emphasizing its association with self-judgment and confidence, particularly in the face of new challenges [21]. There have been few studies that have reported on self-efficacy in nurses and their willingness to provide spiritual care. Recent researches support that nurses with higher levels of self-efficacy tend to perceive themselves as more competent in providing spiritual care [9, 22]. Furthermore, the introduction of a training course focused on spiritual care had a significant impact on nursing students’ self-efficacy in providing spiritual care [10]. This evidence reveals that spiritual care and self-efficacy are highly correlated. Our study demonstrated that self-efficacy is a determinant of one’s willingness to provide spiritual care. Hospital managers should always be seeking ways to enhance the self-efficacy of their nursing staff, as it may result in a higher willingness for them to provide spiritual care.

Attitudes toward spiritual care play a significant role in how nurses perceive and engage with their own spiritual health, commitment to their profession, and caring for patients [23]. Spiritual care competence had a positive relationship with both spiritual well-being and spiritual care perspective among 109 nurses working in intensive care units in Iran [24]. Another study in Iran also discovered that a nurse’s spiritual well-being was found to have a positive relationship with their attitude to spiritual care [25]. These results indicate that it is important to enhance the spiritual well-being of the nursing staff in order to have a better quality of spiritual care. Another past study revealed that a spiritual care program consisting of eight sessions involving four relationship-based domains for oncology nurses had a positive impact on the spiritual well-being [26]. A systematic review reveals educational programs that enhance spiritual intelligence in nurses and nursing students, leading to improved outcomes such as communication skills, job satisfaction, and spiritual care competence [27]. Our study indicated a correlation between spiritual well-being and the willingness to provide spiritual care. Furthermore, SIWB-C played a crucial role in delivering spiritual care among nurses, even though educational programs did not specifically target it. This could be attributed to the various components of subjective well-being, such as positive affect, low levels of negative affect, satisfaction with work or other domains, and life satisfaction [15]. Such findings were particularly evident in our study through the life scheme domain of SIWB-C.

### Limitations of the study

There were certain limitations to the study. Firstly, the study was only conducted among Taiwanese nurses, so the external validity of the study needed careful assessment. Secondly, the willingness to provide spiritual care may have been influenced by physical and/or psychological burnout, factors not considered in this study. Thirdly, it is important to note that the sample predominantly consisted of female participants. As a result, the findings may not be fully generalizable to populations with different gender distributions, and gender-related factors could potentially confound the results. Future studies should explore the relationship between burnout and the willingness to provide spiritual care among nursing staff.

## Conclusion

Our study indicated that both self-efficacy and spiritual well-being were influential factors in determining the provision of spiritual care. Hospital managers should have developed strategies and programs specifically designed to enhance the self-efficacy of their nursing staff. Simultaneously, the effectiveness of any continuing educational programs should have been carefully monitored.

## Data Availability

The datasets utilized and analyzed in this study are accessible upon reasonable request from the corresponding authors.

## Abbreviations

CVI: Content validity index
GSES: General self-efficacy scale
SCNI: Spiritual care needs inventory
SD: Standard deviation
SIWB: Spiritual index of well-being
SIWB-C: Spiritual index of well-being Chinese version
